# Gut Microbiota and Host Thermoregulation in Response to Ambient Temperature Fluctuations

**DOI:** 10.1128/mSystems.00514-20

**Published:** 2020-10-20

**Authors:** Saeid Khakisahneh, Xue-Ying Zhang, Zahra Nouri, De-Hua Wang

**Affiliations:** a State Key Laboratory of Integrated Management of Pest Insects and Rodents, Institute of Zoology, Chinese Academy of Sciences, Beijing, China; b CAS Center for Excellence in Biotic Interactions, University of Chinese Academy of Sciences, Beijing, China; Oregon State University

**Keywords:** food intake, gut microbiome, intermittent temperature, resting metabolic rate, thyroid hormones

## Abstract

Ambient temperature (*T_a_*) is an important factor in shaping phenotypic plasticity. Plasticity is generally beneficial for animals in adapting to their environments. Gut microbiota are crucial in regulating host physiological and behavioral processes. However, whether the gut microbiota play a role in regulating host phenotypic plasticity under the conditions of repeated fluctuations in environmental factors has rarely been examined. We used intermittent *T_a_* acclimations to test the hypothesis that the plasticity of gut microbiota confers on the host a metabolic adaptation to *T_a_* fluctuations. Mongolian gerbils (Meriones unguiculatus) were acclimated to intermittent 5°C to 23°C, 37°C to 23°C or 23°C to 23°C conditions for 3 cycles (totally 3 months). Intermittent *T_a_* acclimations induced variations in resting metabolic rate (RMR), serum thyroid hormones, and core body temperature (*T_b_*). We further identified that the β-diversity of the microbial community varied with *T_a_* and showed diverse responses during the 3 cycles. Some specific bacteria were more sensitive to *T_a_* and were associated with host dynamic metabolic plasticity during *T_a_* acclimations. In addition, depletion of gut microbiota in antibiotic-treated gerbils impaired metabolic plasticity, particularly at low *T_a_*, whereas supplementation with propionate as an energy resource improved the inhibited thermogenic capacity and increased the survival rate in the cold. These findings demonstrate that both gut microbiota and their host were more adaptive after repeated acclimations, and dynamic gut microbiota and their metabolites may confer host plasticity in thermoregulation in response to *T_a_* fluctuations. It also implies that low *T_a_* is a crucial cue in driving symbiosis between mammals and their gut microbiota during evolution.

**IMPORTANCE** Whether gut microbiota play a role in regulating host phenotypic plasticity in small mammals living in seasonal environments has rarely been examined. The present study, through an intermittent temperature acclimation model, indicates that both gut microbiota and their host were more adaptive after repeated acclimations. It also demonstrates that dynamic gut microbiota confer host plasticity in thermoregulation in response to intermittent temperature fluctuations. Furthermore, low temperature seems to be a crucial cue in driving the symbiosis between mammals and their gut microbiota during evolution.

## Abstract (precis)

Whether gut microbiota play a role in regulating host phenotypic plasticity in small mammals living in seasonal environments has rarely been examined. The present study, through an intermittent temperature acclimation model, indicates that both gut microbiota and their host were more adaptive after repeated acclimations. It also demonstrates that dynamic gut microbiota confer host plasticity in thermoregulation in response to intermittent temperature fluctuations. Furthermore, low temperature seems to be a crucial cue in driving the symbiosis between mammals and their gut microbiota during evolution.

## INTRODUCTION

Phenotypic plasticity is a vital adaptation of organisms to complex, variable environments, and it determines the fitness and distribution range of species (1, 2). Ambient temperature (*T_a_*) is an important factor for shaping phenotypic plasticity in small mammals in the temperate and Arctic regions, which are characterized by fluctuating *T_a_* (3). A critical physiological adaptive strategy to cope with *T_a_* fluctuations for small mammals is a changing metabolic rate (4, 5). Small mammal species distributed in different latitudes and habitats exhibit diverse phenotypic variations in response to high and low *T_a_* values. For example, desert rodents usually have a wide thermal neutral zone (TNZ) to cope with winter or cold conditions (5, 6). Hibernating mammals reduce metabolic rate and hibernate to survive the cold season (7). External environment cues, such as *T_a_* and/or photoperiod, can be perceived and transformed into neuronal signaling and induce changes in thyroid hormones and leptin that can meditate metabolic adjustments (8, 9).

Increasing evidence indicates that microorganisms residing in the gut have essential metabolic and immunological functions for the adaptation of their host species (10). It has been demonstrated previously that changes in relative abundances of some microbial taxa can change digestibility of the diet and the amount of energy harvested for the host species (11–13). Additionally, bacterial metabolites act as paracrine or endocrine factors and have a marked effect in regulating energy metabolism in host species (14). Various factors such as season, altitude, diet, and photoperiod have been reported to have an impact on the diversity of gut microbial communities in small mammals. For example, marked seasonal fluctuations in microbial communities were observed in wild mice (15) and hibernating ground squirrels (Ictidomys tridecemlineatus and Urocitellus parryii) on an annual basis (7, 16). However, the effects of increased or decreased *T_a_* on gut microbial community structure were reported mainly in invertebrates or ectotherms (17, 18). Only a few studies have reported cold-induced variation in gut microbial communities in laboratory mice and Brandt’s voles (Lasiopodomys brandtii) (19–21). There are no data on the response of gut microbiota and their relationship with metabolic plasticity in mammal species exposed to frequent high or low *T_a_* in the context of global climate change.

The Mongolian gerbil (Meriones unguiculatus), a rodent species in semiarid steppes, desert grasslands, and agricultural fields of northern China, Mongolia, and the Trans-Baikal region of Russia, faces high fluctuations in annual *T_a_* (range from −47.5°C to 35.3°C) in its habitat and possesses a wide TNZ (26.5°C to 38.9°C) (22). These gerbils exhibited seasonal physiological plasticity in energy intake, resting metabolic rate (RMR), and nonshivering thermogenesis (NST) in the face of seasonal environmental changes, particularly in *T_a_* (4, 23). They also show seasonal behavioral plasticity, such as breeding and food hoarding in natural habitats (24, 25). It was reported that gut microbiota were involved in cold- or huddling-induced thermoregulation in wild small mammals (20). However, we have a major knowledge gap about whether the gut microbiota play a role in beneficial phenotypic plasticity. We used the intermittent *T_a_* acclimation to test the hypothesis that the plasticity in the gut microbiota confers host metabolic adaptation to *T_a_* fluctuations. We first examined the effects of periodic high- and low-*T_a_* acclimation on the diversity and composition of the gut microbial community, as well as the corresponding changes in thyroid hormones and metabolic regulation. In addition, we determined the role of the gut microbiota in conferring host metabolic benefit for survival at a high or low *T_a_*.

## RESULTS

### Dynamic changes in metabolic phenotypes during intermittent high- and low-*T_a_* acclimations.

**FIG 1:**
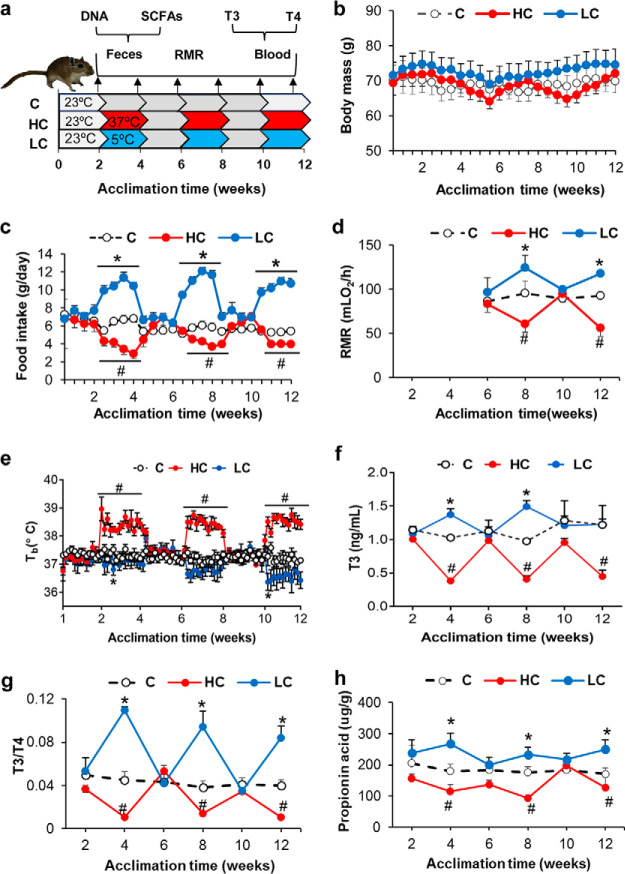
The plasticity of metabolic phenotypes and metabolites during intermittent temperature acclimations in Mongolian gerbils. (a) Schematic overview of experimental design. (b) Body mass monitoring. (c) Food intake. (d) Resting metabolic rate (RMR). (e) Core body temperature (*T_b_*). (f, g) Serum T3 and the T3/T4 ratio. (h) Concentration of propionic acid. Data are presented as means ± standard error of the mean (SEM). *, *P* < 0.05 for LC versus control. *#*, *P* < 0.05 for HC versus control.

To test the responses to intermittent temperature variations, adult Mongolian gerbils were acclimated to intermittent 23°C to 23°C (C), 37°C to 23°C (HC) and 5°C to 23°C (LC) conditions (26) (Fig. 1a). Body mass was not influenced by intermittent *T_a_* manipulation [F_(2, 20)_ = 0.565, *P* = 0.577; Fig. 1b]. Food intake increased (by 60% in the first and 57% in the last exposure) in LC gerbils and decreased (by 53% in the first and 38% in the last exposure) in the HC group compared to the control group [F_(2, 24)_ = 34.927, *P* < 0.001; Fig. 1c]. RMR increased by 30% in the 8th week and by 27% in the 12th week in LC and decreased by 37% in the first and 39% in the last in HC [8th week, F_(2, 12)_ = 4.494, *P* = 0.035; 12th week, F_(2, 12)_ = 4.652, *P* = 0.032] (Fig. 1d). Core body temperature (*T_b_*) fluctuated with time [F_(80, 640)_ = 4.145, *P* < 0.001] and was affected by group [F_(2, 8)_ = 10.231, *P* = 0.006] and the interaction of time and group [F_(160, 640)_ = 10.770, *P* < 0.001; Fig. 1e]. *T_b_* values were 1.3°C higher in the gerbils exposed to the 3rd period of 37°C and 0.7°C lower in those exposed to the 3rd period of 5°C than in 23°C (Fig. 1e). Both T3 and the T3/T4 ratio increased in the periods of 5°C and decreased in the periods of 37°C, and they returned to control levels after acclimation to 23°C (Fig. 1f and g). The propionic acid concentration increased in LC and decreased in HC compared with the control in the 4th [F_(2, 27)_ = 7.622, *P* = 0.003], 8th [F_(2, 21)_ = 10.859, *P* = 0.001], and 12th weeks [F_(2, 22)_ = 5.020, *P* = 0.017], and returned to control levels in the 6th [F_(2, 22)_ = 2.223, *P* = 0.133] and 10th weeks [F_(2, 22)_ = 0.640, *P* = 0.538; Fig. 1h]. Other short-chain fatty acids (SCFAs) did not differ among groups during acclimation (see Table S1 in the supplemental material).

10.1128/mSystems.00514-20.4 TABLE S1 Changes in short-chain fatty acids (SCFAs; μg/g) during intermittent temperature acclimations. Data are presented as means ± SEM. Different superscript letters indicate significant differences among groups (*P* < 0.05). C, maintained at 23°C; HC, acclimated to periodic 37° and 23°C; LC, acclimated to periodic 5°C and 23°C. Download Table S1, DOCX file, 0.02 MB. Copyright © 2020 Khakisahneh et al. 2020 Khakisahneh et al. This content is distributed under the terms of the Creative Commons Attribution 4.0 International license.

RMR was correlated positively with food intake and T3/T4 during intermittent high-*T_a_* acclimation (in the HC group but not in the LC or C groups; see Tables S2 to S^4^ and Fig. S1 in the supplemental material), and correlated positively with the concentrations of propionic acid, acetic acid, butyric acid, and valeric acid during intermittent low-*T_a_* acclimation (Table S4; Fig. S1). The T3/T4 ratio was correlated positively with food intake and propionic acid concentration during intermittent high- and low-*T_a_* acclimations (Tables S3 and S4; Fig. S1).

10.1128/mSystems.00514-20.1 FIG S1 Pearson correlation between physiological parameters. Correlations between resting metabolic rate (RMR) and (a) food intake, (b) T3/T4 ratio, (c) propionic acid, and (d) acetic acid, and between T3/T4 ratio and (e) food intake and (f) propionic acid. C, maintained at 23°C; HC, acclimated to periodic 37° and 23°C; LC, acclimated to periodic 5°C and 23°C. Download FIG S1, TIF file, 0.6 MB. Copyright © 2020 Khakisahneh et al. 2020 Khakisahneh et al. This content is distributed under the terms of the Creative Commons Attribution 4.0 International license.

10.1128/mSystems.00514-20.5 TABLE S2 Correlations between physiological parameters and short-chain fatty acids (SCFAs) at 23°C. *, Correlation is significant at the 0.05 level (2-tailed). **, Correlation is significant at the 0.01 level (2-tailed). Download Table S2, DOCX file, 0.01 MB. Copyright © 2020 Khakisahneh et al. 2020 Khakisahneh et al. This content is distributed under the terms of the Creative Commons Attribution 4.0 International license.

10.1128/mSystems.00514-20.6 TABLE S3 Correlations between physiological parameters and short-chain fatty acids (SCFAs) during intermittent 37°C acclimations. *, Correlation is significant at the 0.05 level (2-tailed). **, Correlation is significant at the 0.01 level (2-tailed). Download Table S3, DOCX file, 0.02 MB. Copyright © 2020 Khakisahneh et al. 2020 Khakisahneh et al. This content is distributed under the terms of the Creative Commons Attribution 4.0 International license.

10.1128/mSystems.00514-20.7 TABLE S4 Correlations between physiological parameters and short-chain fatty acids (SCFAs) during intermittent 5°C acclimations. *, Correlation is significant at the 0.05 level (2-tailed). **, Correlation is significant at the 0.01 level (2-tailed). Download Table S4, DOCX file, 0.02 MB. Copyright © 2020 Khakisahneh et al. 2020 Khakisahneh et al. This content is distributed under the terms of the Creative Commons Attribution 4.0 International license.

### Dynamic microbial diversity during intermittent high- and low-*T_a_* acclimations.

**FIG 2:**
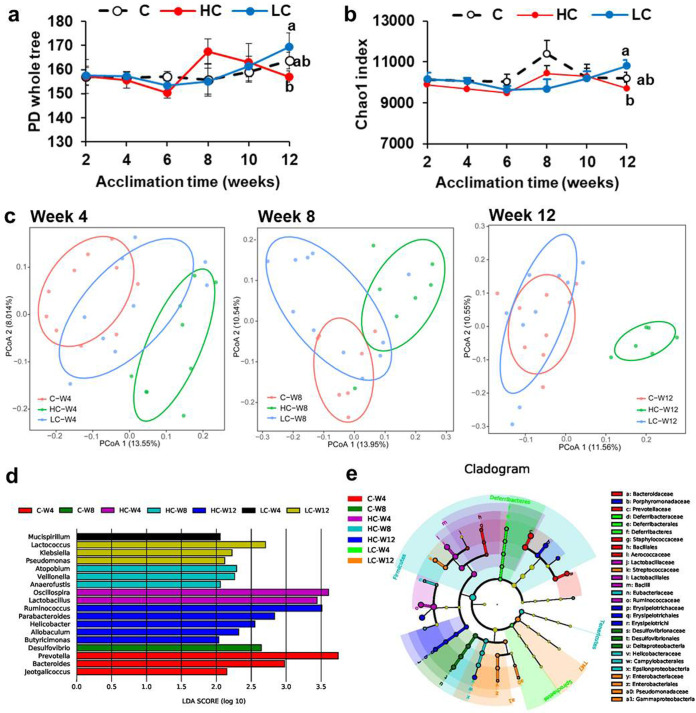
Dynamic changes in the diversity and structure of gut microbial community during intermittent temperature acclimations. (a) Phylogenetic diversity (PD) whole-tree analysis and (b) Chao1 index for the samples. Different letters indicate significant differences among groups (*P* < 0.05). (c) Principal coordinate analysis (PCoA) based on Bray-Curtis distance between the samples in weeks 4, 8, and 12 of *T_a_* acclimation. (d) The plot was generated using the LEfSe tool. The bar plots represent the significantly differential taxa at the genus level among groups, based on effect size (LDA score [log 10], >2). (e) Cladogram representing taxa at the genus level enriched in fecal microbial community of the groups detected by the LEfSe tool. The circle’s diameter is proportional to the taxon’s abundance.

To identify whether the gut microbiota exhibited dynamic variations in response to intermittent-*T_a_* acclimation, we analyzed 16S rRNA gene sequences from fecal samples at different time points. The sequencing resulted in a total of 4,887,962 valid reads and identified 107,420 unique operational taxonomic units (OTUs) at a threshold of 97% sequence identity. The sequence number per sample was 31,740 ± 1,640. The rarefaction curve of Goods coverage for all samples reached saturation (see Fig. S2 in the supplemental material), indicating that most bacteria were identified in this study. The diversity and richness of gut microbiota (α-diversity) fluctuated during intermittent temperature treatment (Fig. 2a and b; see also Table S5 in the supplemental material), but evident group differences were found in the 3rd acclimation period (12th week), with significant increases in phylogenetic diversity (PD) whole-tree and Chao1 levels in the LC compared to those in the HC group (Fig. 2a and b and Table S5). The principal-coordinate analysis (PCoA) graphs clearly illustrated separation of the microbial community (β-diversity) among different groups, especially in the first period (4th week, analysis of similarity [ANOSIM], *R* = 0.384, *P* = 0.001) and second period (8th week, ANOSIM, *R* = 0.248, *P* = 0.001) of acclimation at 5°C or 37°C. After the third (12th week, ANOSIM, *R* = 0.397, *P* = 0.001) intermittent acclimation, β-diversity of the HC (37°C) group showed a complete separation from the control group (C) (23°C) and LC (5°C) groups, whereas the clusters of the latter two groups overlapped (Fig. 2c). The linear discriminant analysis (LDA) effect size (LEfSe) method with an LDA score of >2 identified differential biomarkers at the genus level in fecal microbial community of different groups in weeks 4, 8, and 12 of *T_a_* acclimation (Fig. 2d and e).

10.1128/mSystems.00514-20.2 FIG S2 The rarefaction curve of Goods coverage for all fecal samples. Download FIG S2, TIF file, 0.06 MB. Copyright © 2020 Khakisahneh et al. 2020 Khakisahneh et al. This content is distributed under the terms of the Creative Commons Attribution 4.0 International license.

10.1128/mSystems.00514-20.8 TABLE S5 Alpha diversity, including Chao1, observed OTUs, Shannon index, and phylogenetic diversity (PD) whole-tree in different groups under intermittent temperature acclimations. Data are presented as means ± SEM. Different superscript letters indicate significant differences among groups (*P* < 0.05). C, maintained at 23°C; HC, acclimated to periodic 37° and 23°C; LC, acclimated to periodic 5°C and 23°C. Download Table S5, DOCX file, 0.02 MB. Copyright © 2020 Khakisahneh et al. 2020 Khakisahneh et al. This content is distributed under the terms of the Creative Commons Attribution 4.0 International license.

**FIG 3:**
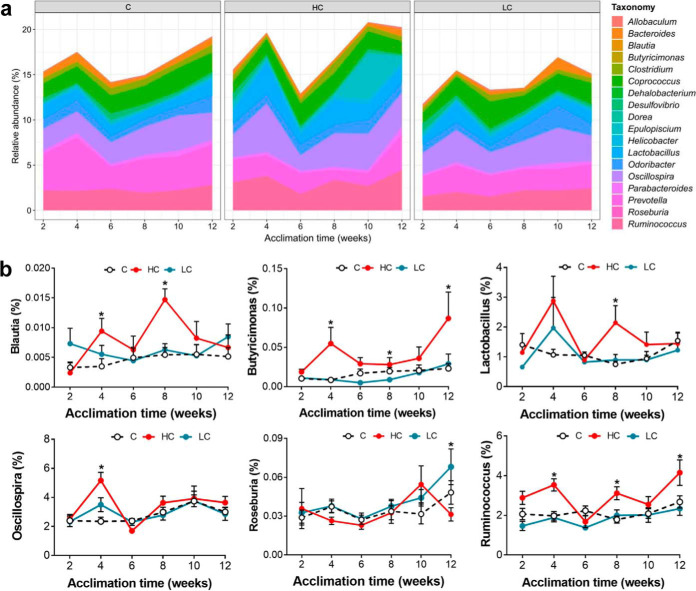
Dynamic changes in the proportion of specific bacteria under intermittent high and low ambient temperature (*T_a_*). (a) The fluctuating patterns of different taxa of the top 18 representative genera during intermittent acclimation in C (23°C to 23°C), HC (37°C to 23°C), and LC (5°C to 23°C) groups. (b) Relative abundance of different bacteria at the genus level during the acclimation time. Data are presented as means ± SEM. *, *P* < 0.05 versus control.

Fluctuating patterns of relative abundances of the top 18 representative genera were observed, particularly for the HC group during intermittent acclimation (Fig. 3a). The relative abundances of Blautia and Lactobacillus spp. increased in both the first and second periods at 37°C acclimation compared to the control (*P* < 0.001; Fig. 3b). The relative abundances of Butyricimonas and Ruminococcus spp. were higher during all the periods of 37°C acclimation (*P* < 0.001; Fig. 3b). The relative abundance of Oscillospira increased significantly only in the first time of 37°C acclimation compared to the control [F_(2, 25)_ = 10.136, *P* = 0.001], and showed no change in 5°C-exposed gerbils (Fig. 3b). The relative abundance of Roseburia increased at 5°C only in the third period of acclimation [12th week, F_(2, 21)_ = 3.978, *P* = 0.036; Fig. 3b].

**FIG 4:**
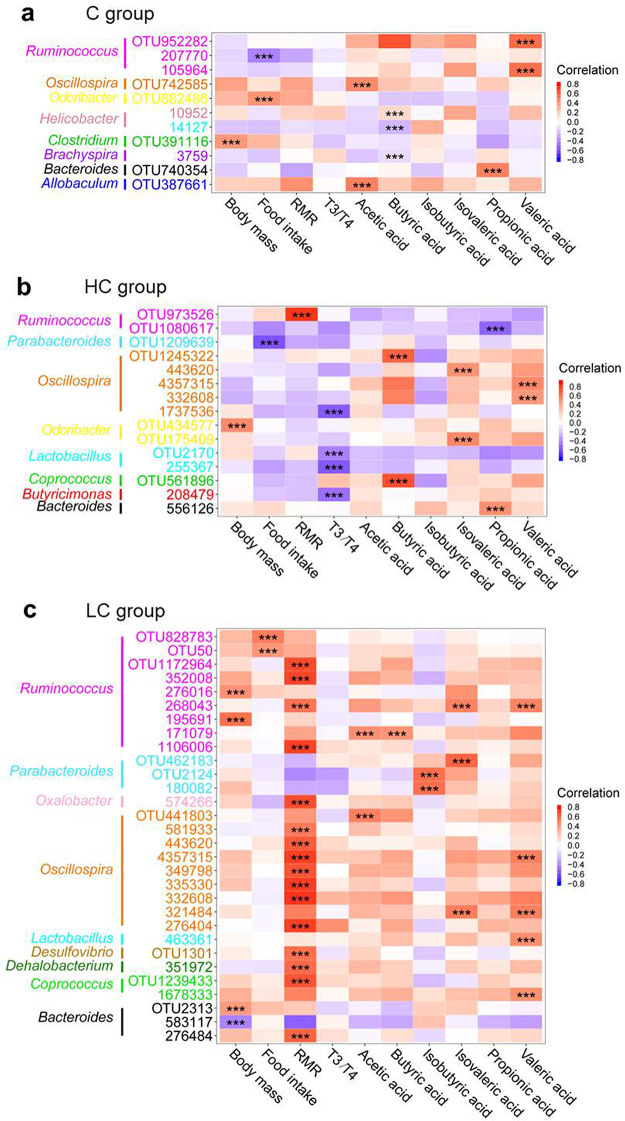
Heatmap of correlations between operational taxonomic units (OTUs) and physiological parameters. (a) Pearson correlation in the C group (23°C to 23°C), (b) Pearson correlation in the HC group (37°C to 23°C), (C) Pearson correlation in the LC group (5°C to 23°C). The OTU identifiers (IDs) with only numbers were from the Greengenes database, whereas those with the letters were clustered into *de novo* OTUs. ***, false-discovery rate (FDR)-corrected *P* < 0.001.

We further analyzed the correlations between bacterial taxa and metabolic phenotypes by Pearson correlation analyses for the control (Fig. 4a), HC (Fig. 4b), and LC (Fig. 4c) groups during all acclimation periods. Some specific bacteria, such as Butyricimonas, *Lactobacillus*, and *Oscillospira* were correlated negatively with the T3/T4 ratio, and Parabacteroides was correlated negatively with food intake during intermittent 37°C acclimation (Fig. 4b). The bacterial taxa of Coprococcus, Dehalobacterium, Desulfovibrio, *Oscillospira*, and *Ruminococcus* were correlated positively with RMR, and the taxa of *Ruminococcus* were correlated positively with food intake under intermittent 5°C acclimation (Fig. 4c).

### Metabolic phenotypes and gut microbial diversity in antibiotic-treated gerbils in response to high or low *T_a_*.

**FIG 5:**
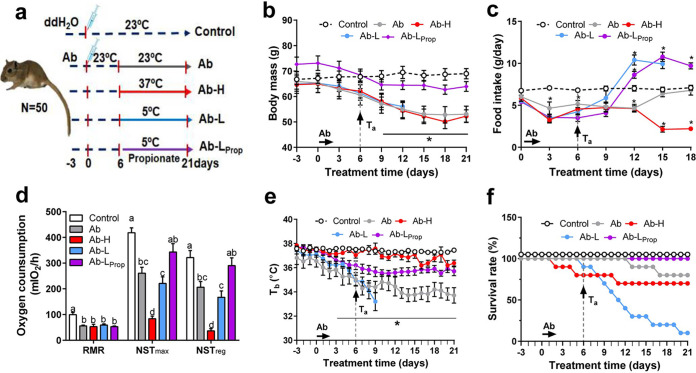
Energy metabolism, body temperature and survival rate in antibiotic-treated gerbils during different *T_a_* acclimations. (a) Schematic overview of experimental design. (b) Body mass. (c) Food intake. (d) Resting metabolic rate (RMR), maximum nonshivering thermogenesis (NST_max_) and regulatory NST (NST_reg_). (e) Core body temperature (*T_b_*). (f) Survival rate. Data are presented as means ± SEM. *, *P* < 0.05, Ab, Ab-H, and Ab-L versus control (b), Ab, Ab-H, Ab-L, and Ab-L_Prop_ versus control (c), or Ab, Ab-L, and Ab-L_Prop_ versus control (e). Different letters above columns indicate significant differences among groups (*P* < 0.05).

To verify the role of gut microbiota for host defense in low or high *T_a_*, an antibiotic cocktail was administered via intragastric gavage, and the animals were acclimated to high or low *T_a_* (Fig. 5a). Following antibiotic administration, body mass dropped in gerbils except in those that received propionate (Ab-L_Prop_ group) [F_(4, 33)_ = 3.037, *P* = 0.031; Fig. 5b]. Food intake was lower in antibiotic recipients than in the control group [F_(4, 29)_ = 7.549, *P* < 0.001; Fig. 5c]. During 5°C acclimation, food intake was 77% higher in the Ab-L and 111% higher in the Ab-L_Prop_ groups than their initial levels before *T_a_* acclimation, and it was 54% lower in the gerbils acclimated to 37°C [F_(6, 132)_ = 2.485, *P* = 0.026; Fig. 5c]. Compared to the control group, all gerbils that were treated with antibiotics reduced RMR by 45% [F_(4, 25)_ = 6.375, *P* = 0.001; Fig. 5d]. Treatment with antibiotics resulted in significant reductions in the maximum nonshivering thermogenesis (NST_max_) [F_(4, 25)_ = 10.484, *P* < 0.001] and the regulatory NST (NST_reg_) [F_(4, 25)_ = 9.929, *P* < 0.001]. During cold stimulation, the Ab-L_Prop_ gerbils had higher NST_max_ and NST_reg_ values than the Ab-L group (Fig. 5d). *T_b_* decreased from 36.8°C ± 0.6°C to 34.2°C ± 0.6°C after 1 week of antibiotic treatment and then remained stable (Fig. 5e). The Ab-H gerbils had the same *T_b_* as the control (*P* > 0.05), but the Ab-L gerbils reduced *T_b_* to 33.2°C ± 0.7°C 4 days after exposure to 5°C [F_(4, 18)_ = 14.01, *P* < 0.001; Fig. 5e], and the survival rate dropped to 50% 1 week after cold exposure (Fig. 5F). However, the Ab-L_Prop_ gerbils maintained a stable *T_b_* (35.8°C ± 0.4°C), and no animal died during cold acclimation (Fig. 5e and f).

**FIG 6:**
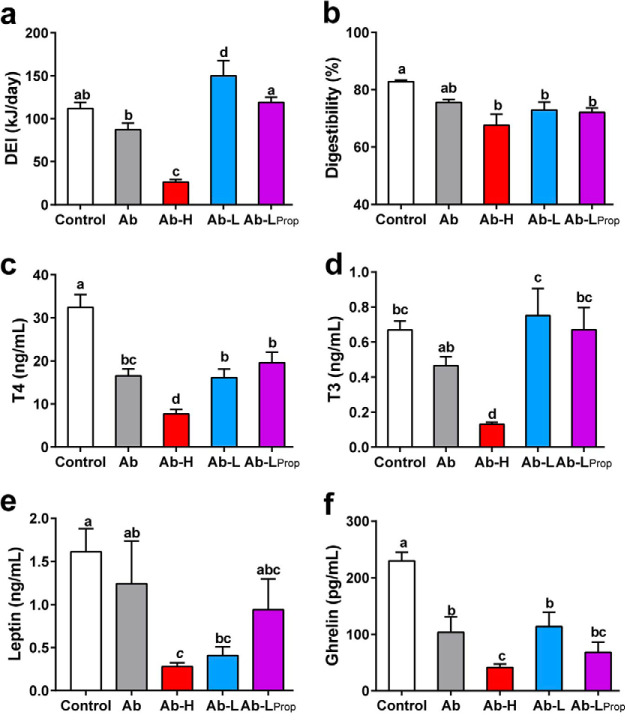
Energy intake and serum hormones in antibiotic-treated gerbils at different *T_a_* values. (a) Digestible energy intake (DEI). (b) Digestibility. (c to f) Serum T3, T4, leptin, and ghrelin levels. Data are presented as means ± SEM. Different letters above columns indicate significant differences among groups (*P* < 0.05).

Following antibiotic treatment, digestible energy intake (DEI) differed among groups [F_(4, 29)_ = 25.312, *P* < 0.001; Fig. 6a; see also Table S6 in the supplemental material]. DEI increased in the Ab-L group (*post hoc*, *P* < 0.001) and decreased in the Ab-H gerbils (*post hoc*, *P* < 0.001) compared with that in the Ab animals, and DEI in the Ab gerbils supplemented with propionate at 5°C (Ab-L_prop_) was lower than that in the Ab-L group (*P* = 0.015). Antibiotic treatment independent of *T_a_* led to a reduced diet digestibility [F_(4, 29)_ = 5.641, *P* = 0.002; Fig. 6b]. Serum T3 levels also differed among groups [F_(4, 28)_ = 10.706, *P* < 0.001; Fig. 6c]. Ab-L gerbils had higher (*P* = 0.002) and Ab-H had lower (*P* = 0.017) serum T3 levels than the Ab group. Serum T4 levels were lower in all antibiotic-treated groups [F_(4, 28)_ = 20.896, *P* < 0.001; Fig. 6d]. Serum leptin levels were lower in the Ab-L and Ab-H than the control and Ab groups [F_(4, 24)_ = 4.477, *P* = 0.008; Fig. 6e]. Serum ghrelin levels were lower in all antibiotic-treated groups than in the control gerbils [F_(4, 28)_ = 14.780, *P* < 0.001; Fig. 6f].

10.1128/mSystems.00514-20.9 TABLE S6 Effect of antibiotic treatment on digestible energy intake (DEI), diet digestibility, and concentrations of serum T3, T4, leptin, and ghrelin. Data are presented as means ± SEM. Different superscript letters indicate significant differences among groups (*P* < 0.05). Control, maintained at 23°C without any treatment. Ab, received antibiotics at 23°C; Ab-H, received antibiotics and were acclimated to 37°C; Ab-L, received antibiotics and were acclimated to 5°C; Ab-L_Prop_, received antibiotics and propionate and were acclimated to 5°C. Download Table S6, DOCX file, 0.01 MB. Copyright © 2020 Khakisahneh et al. 2020 Khakisahneh et al. This content is distributed under the terms of the Creative Commons Attribution 4.0 International license.

Antibiotic treatment led to reductions both in the diversity (see Fig. S3a and b and Table S7 in the supplemental material) and relative abundance (Fig. S3c to e) of gut microbial communities. At the phylum level, the proportions of Firmicutes taxa decreased from 34% in the control group to 4% to 14% in all antibiotic recipients acclimated to different *T_a_*, but Proteobacteria taxa increased from only 7% in the control to 65% to 90% in the antibiotic-treated groups (Fig. S3c and d). At the genus level, the relative abundances of *Lactobacillus* [F_(4, 26)_ = 13.943, *P* < 0.001] and *Oscillospira* [F_(4, 26)_ = 31.718, *P* < 0.001] reduced after antibiotic treatment (Fig. S3e). Furthermore, at the species level, the relative abundances of Bifidobacterium adolescentis in Ab-H and Morganella morganii in Ab-L increased after antibiotic treatment and different *T_a_* value (*P* < 0.01, Fig. S3e).

10.1128/mSystems.00514-20.3 FIG S3 The dynamic diversity and structure of gut microbial community in antibiotic-treated gerbils during different ambient temperature (*T_a_*) acclimations. (a) The α-diversity analysis indicated by Shannon index and observed operational taxonomic units (OTUs) for the samples. (b) Principal coordinate analysis (PCoA) plots of Bray-Curtis distance matrix in each sample. (c) Relative abundance of different bacteria at the phylum level in fecal microbial community. (d) Fraction of phylum. (e) Relative abundance of different bacteria at genus and species levels. Bifidobacterium adolescentis, B. adolescentis; M. morganii, Morganella morganii. Download FIG S3, TIF file, 0.8 MB. Copyright © 2020 Khakisahneh et al. 2020 Khakisahneh et al. This content is distributed under the terms of the Creative Commons Attribution 4.0 International license.

10.1128/mSystems.00514-20.10 TABLE S7 Alpha diversity, including Chao1, observed OTUs, Shannon index, and PD whole tree, in antibiotic-treated gerbils during different *T_a_* acclimations. Data are presented as means ± SEM. Different superscript letters indicate significant differences among groups (*P* < 0.05). Control, maintained at 23°C without any treatment. Ab, received antibiotics and at 23°C; Ab-H, received antibiotics and were acclimated to 37°C; Ab-L, received antibiotics and were acclimated to 5°C; Ab-L_Prop_, received antibiotics and propionate and were acclimated to 5°C. Download Table S7, DOCX file, 0.01 MB. Copyright © 2020 Khakisahneh et al. 2020 Khakisahneh et al. This content is distributed under the terms of the Creative Commons Attribution 4.0 International license.

## DISCUSSION

The annual cycle in thermal physiology of a small mammal is affected by photoperiod, *T_a_*, and food availability (6, 26). In the present study, we observed that the plasticity of host thermoregulation was associated with dynamic changes in gut microbial profiles in response to repeated high or low *T_a_*. Furthermore, we illustrated that gut microbiota depletion could inhibit metabolic plasticity and affect survival in cold-exposed gerbils.

Seasonal variations in thermal physiology can maintain stable body temperature and ensure survival of small mammals living in the temperate and Arctic regions. Most previous studies used a single manipulation or one period of acclimation to test the phenotypic plasticity of small mammals in response to changes in environmental cues (4, 5, 27). However, phenotypic plasticity may include many other factors besides environmental cues *per se*, including stress, and also, with only one period of acclimation it is difficult to determine whether the acclimation is beneficial or not. In this study, we established an intermittent *T_a_*-acclimated model and found that the gerbils exhibited increases in food intake, RMR, and the T3/T4 ratio under low *T_a_* and exhibited decreases in these variables under high *T_a_*. Small mammals increased food intake for the high energy expenditure in the cold and kept body mass relatively stable (4, 5). The positive correlations between thyroid hormones and RMR or food intake during repeated cold or hot acclimations supported the involvement of thyroid hormones in thermoregulation in small mammals. In contrast, the gerbils reduced food intake and RMR, but still had a higher *T_b_* during hot acclimation. The gerbils decreased food intake by 53% in the first acclimation at 37°C but by 38% in the last acclimation, suggesting that acclimation experience is beneficial to the animals to tolerate extreme heat. These phenotypic data suggest that repeated acclimations induced beneficial consequences for small mammals, especially for thermal tolerance to extreme high *T_a_*.

Microbial diversity is the result of coevolution between the microbial communities and their hosts and is shaped by both genetic and environmental factors (28). In wild mammals, studies have shown that gut microbial diversity varies with seasons, photoperiod, temperature, food, altitude, geography, and social interaction (15, 20, 29). The α-diversity of the microbial community increased at a low *T_a_* and decreased at a high *T_a_* during the last period of acclimation. The increase in the Chao1 index of the control group at week 8 was unexpected, which may be affected by some contamination during sample collection. The β-diversity showed variations, with both high and low *T_a_* during the first and second acclimations but not during the third cold acclimation. Similar patterns were also observed in the relative abundances of *Firmicutes*, Bacteroidetes, *Blautia*, and *Oscillospira*. Additionally, the relative abundances of Butyricimonas and *Ruminococcus* spp. always increased with intermittent high *T_a_*, indicating that microbes from these genera may be more sensitive to hot condition. These genera are involved in important metabolic functions in hosts and can also affect immunity maintenance and anti-inflammatory properties (30). During low-*T_a_* acclimation, the bacterial diversity increased. The changes in bacteria may contribute to regulating a distinct set of hormones, such as peptide YY and glucagon-like peptide 1 from the gut and adiponectin secretion from adipocytes, to control food intake and support the high energy demands of the host (31, 32). After repeated cold acclimations, the bacterial β-diversity overlapped with that of the control group, and all genera except for Roseburia showed no obvious changes in low-*T_a_*-induced gerbils, suggesting that the gerbils and their gut microbiota have evolved to be more adaptive to low *T_a_*.

The dynamic variations in gut microbiota occurred in parallel with periodic variations in food intake, RMR, thyroid hormones, and SCFAs (especially propionic acid). Diet (amount and macronutrient composition) is one of the major drivers of microbiota abundance and taxonomic composition (33). Therefore, the increase in food intake during cold acclimation may be one reason leading to changes in gut microbiota. However, our previous study, via a paired-feeding protocol (the amount of food intake in cold-acclimated animals was restricted to the same level as that of the control in the warm condition), concluded that low temperature *per se* led to microbiota differences, which were not due to overfeeding at a low *T_a_* (21). The *T_b_* of gerbils increased by 1.3°C when acclimated repeatedly to high *T_a_*, and decreased by 0.7°C when acclimated repeatedly to low *T_a_*. The previous study also showed that Brandt’s voles reduced *T_b_* by 1°C in response to low-*T_a_* acclimation (20). It indicated that small mammals would adjust their *T_b_* to a new set point to adapt to the low or high *T_a_*. Factors such as temperature and pH may shape gut microbial community by mediating microbe-microbe interactions (34, 35). Consequently, blooms of specific bacteria may modulate host metabolic rate and thus affect *T_b_*.

The role of gut microbiota in contributing to host metabolic and thermal plasticity was further confirmed in antibiotic-treated gerbils. There are no germfree wild animals, so we used composite antibiotics to deplete gut microbiota. The microbial data showed that bacterial α-diversity indicated by the Shannon index was 58% lower in antibiotic-treated gerbils than that their control counterparts. Depletion of gut microbiota led to a reduced metabolic plasticity of the host, with reductions in RMR and NST. The gerbils with depleted microbiota could only maintain a lower *T_b_* (34.2°C versus 36.8°C) at room temperature and, when they were exposed to 5°C, could not survive due to continuous drops in *T_b_*. The inability to regulate thermogenesis in the gerbils without a sufficient gut microbial community may be related to reduced digestibility and less energy acquisition from bacterial fermentation. This possibility was supported by the evidence that supplementation of propionate to antibiotic-treated gerbils led to increases in the regulatory NST, and the propionate recipients could keep a stable *T_b_* and thus survive at 5°C. In support of our result, other studies in mice indicated that gut microbiota and their metabolites contribute to regulating thermogenesis under cold conditions (19, 36). The mechanism underlying the interactions between microbes and metabolism should be further examined.

For the first time, our study illustrated the role of gut microbiota in controlling host phenotypic plasticity in the face of fluctuating *T_a_*. Both the hosts and their symbiotic microbiota were more adaptive to high or low *T_a_* after repeated exposures. Using antibiotic-treated gerbils, we demonstrated the reduced function of the gut microbial community and reduced digestibility and thermogenesis of the host. These gerbils could not survive at a low *T_a_*. The situation could be reversed by supplement of propionate as an energy resource. The intermittent-*T_a_* acclimation model not only mimics seasonal environments but also provides an effective approach to promote individual tolerance to extreme *T_a_* and population fitness in fluctuating environments. Further studies are needed to determine how the endogenous gut microbiota perceive the thermal environment and alter the community to benefit both sides of the holobiont in endothermic mammals.

## MATERIALS AND METHODS

### Experimental animals.

Mongolian gerbils were housed with same-sex siblings in plastic cages (30 × 15 × 20 cm) with sawdust bedding. Every 3 years, the breeding colonies were rejuvenated with wild gerbils from the desert grasslands of Inner Mongolia or with gerbils from other laboratories. All gerbils were maintained at 23°C ± 1°C on a 16:8 light-dark cycle and were offered commercial standard rat pellets (Beijing KeAo Bioscience Co.) and water *ad libitum*. All animals were habituated in individual cages at least 2 weeks before experiments. All procedures in the study were approved by the Animal Care and Use Committee of the Institute of Zoology, Chinese Academy of Sciences.

### Experimental designs.

Experiment 1 tested the effects of intermittent temperature on metabolic rate and gut microbial community. Thirty Mongolian gerbils (between 6 months and 1 year of age) were divided randomly into 3 groups (*n *= 10 per group). In the first 2 weeks, the gerbils were kept at 23°C ± 1°C, then transferred to a high *T_a_* (37°C ± 1°C, HC group) or a low *T_a_* (5°C ± 1°C, LC group) for another 2 weeks, then returned to 23°C ± 1°C; this was done 3 times. Another group was used as a control group (C) and was maintained at 23°C ± 1°C for 12 weeks. The 2-week intervals were chosen based on a previous study by Zhang and Wang (5), which showed that the animals can be acclimated to a new *T_a_* in 2 weeks with stable food intake and thermogenic responses. Body mass (± 0.1 g) and food consumption were measured every 3 days during the course of the experiment. RMR was measured at every 2-week interval. At the end of every 2-week interval, blood was collected from the infraorbital vein for the measurement of thyroid hormones. Fresh feces were collected and immediately frozen in liquid nitrogen and stored at −80°C for the measurement of SCFAs and for DNA extraction.

Experiment 2 examined the role of gut microbiota in host response to thermal fluctuation. Another 50 gerbils were divided into 5 groups (*n *= 10 per group). A control group (with daily gavage of 200 μl deionized water) was maintained at 23°C ± 1°C during the experimental period. The other 4 groups were all administered an antibiotic cocktail (100 mg/ml neomycin, 50 mg/ml streptomycin, 50 mg/ml vancomycin, 100 mg/ml metronidazole, 1 mg/ml bacitracin, 170 mg/ml gentamicin, and 1 mg/ml ampicillin) in 200 μl via intragastric gavage once a day during the whole experiment (19, 37). These antibiotic-treated gerbils were first kept at 23°C ± 1°C for 6 days and then either at 23°C ± 1°C (Ab), or at a high *T_a_* (37°C ± 1°C, Ab-H), at a low *T_a_* (5°C ± 1°C, Ab-L), and at a low *T_a_* together with a daily gavage of propionate at a dose of 250 mg/kg body mass (Ab-L_Prop_) (38). We gavaged the gerbils with propionate based on the changes in SCFAs during intermittent *T_a_* acclimation in experiment 1. Body mass, food intake, and *T_b_* were monitored every 3 days. RMR and NST were measured in the third week of *T_a_* acclimation, and blood was collected from the infraorbital vein for later measurement of serum hormones.

### Core body temperature.

The core body temperature (*T_b_*) of the gerbils was recorded using a Thermochron iButton (catalog no. DS1922L-F5, with a precision of 0.0625°C) (20). Animals were anesthetized via an intraperitoneal injection of pentobarbital sodium (1%) with a dose of 50 mg/kg. The iButton, coated with a thin layer of silicon (Elastostil E41; Wacker) for water protection, was implanted in the abdomen of each gerbil and was programmed to store *T_b_* every 60 min beginning 1 week after implantation. At the end of experiment, the iButton was removed, and all records were read using OneWireViewer Software.

### Metabolic trials.

A multichannel open flow respirometer (TSE LabMaster, Germany) was used for RMR measurement as described previously (20, 39, 40). Briefly, 0.9 liters/min were pumped through the chamber, which was set at 30°C ± 0.5°C (within the TNZ of Mongolian gerbils). The respirometry system was run for 3 h, and RMR was calculated as an average of 3 consecutive and minimum readings of oxygen consumption after 1 h. RMR data from the 2nd to 4th weeks was missed due to the machine’s malfunction.

We injected norepinephrine (NE; Shanghai Harvest Pharmaceutical Co., Ltd.) into each gerbil to induce the NST_max_ (4). The dosage of NE was calculated according to the following equation: NE (mg/kg) = 6.6 × *M_b_*^−0.458^, where *M_b_* is the body mass in grams (41, 42). During a 1-h measurement, gerbils were housed individually in the metabolic chamber with a volume of 2.7 liters (type I for mice) at 25 ± 1°C. The 3 highest consecutive readings of oxygen consumption after 15 to 20 min were averaged to calculate the NST_max_. The regulatory NST (NST_reg_), which is produced from brown adipose tissue (BAT), was calculated by NST_max_ minus RMR (43).

### Energy intake and digestibility.

Food intake was determined by weighing the food offered and the food remains over 3 days. From days 15 to 18, the uneaten food and feces together with the bedding material were collected and then oven-dried at 60°C for at least 72 h. Food and feces were separated manually, then weighed, and the energy content of food and feces were measured using an oxygen bomb calorimeter (IKA C2000; Germany). The bomb calorimeter was calibrated by burning benzoic acid. Gross energy intake (GEI), digestible energy intake (DEI), and digestibility were calculated using the following equations:

GEI (kJ/day) = dry food intake (g/day) × food energy content (kJ/g dry matter)

fecal energy (kJ/day) = dry feces (g/day) × fecal energy content (kJ/g dry matter)

DEI (kJ/day) = GEI (kJ/day) − fecal energy (kJ/day)

digestibility (%) = DEI (kJ/day)/GEI (kJ/day) × 100%

### Serum hormone assays.

The ^125^I radioimmunoassay (RIA) kits of T3 and T4 (Institute of Atomic Energy, Beijing, China) that were previously validated for Mongolian gerbil were used to quantify serum T3 and T4 concentrations. The intra-assay coefficients of variation (CV) were 5.1% for T3 and 4.4% for T4 (44).

Serum ghrelin levels were measured using an enzyme-linked immunosorbent assay (ELISA) kit (catalog no. CEA991Ra; Cloud-Clone Corp.), and serum leptin levels were measured using an ELISA kit for leptin (catalog no. SEA084Ra; Cloud-Clone Corp.) according to the manufacturer’s instructions. Absorbance was measured at 450 nm against a blank using an ELISA reader (RayBiotech, Canada). The intra- and interassay CVs were <10% and <15% for both kits. The minimum detectable dose for ghrelin was 52.3 pg/ml, and that for leptin was 0.129 ng/ml.

### SCFAs.

Six main SCFAs were measured by gas chromatography (GC) (Agilent7890A; Agilent Technologies, Germany) according to protocols that were described before (20, 45). For extraction, fecal sample (0.2 g) was mixed with double-distilled water (ddH_2_O) and centrifuged at 13,000 rpm at 4°C for 20 min. The supernatant was added to H_3_PO_4_ (25%) at a ratio of 9:1, filtered (0.22 μm), and then SCFAs were separated in a 30 m× 0.25 mm× 0.25 μm DB-WAX column (polyethylene glycol 20000; Agilent Technologies) for separation of SCFAs. The system was operated at a maximum temperature of 250°C with helium (>99.999%) as a carrier gas at a constant flow rate of 1 ml/min. Splitless injection of 0.5 μl of sample was done at 230°C. The temperature was programmed at 60°C for 1 min, increased at a rate of 5°C/min to 200°C, and then at 10°C/min to 230°C. For each sample, the total running time lasted 32 min. The SCFAs were identified by comparing their retention times with those of authentic reference compounds and quantified by the abundance relative to that of the standard.

### DNA extraction and 16S rRNA gene sequencing.

Total DNA was extracted from feces (180 to 220 mg) by 2× cetyltrimethyl ammonium bromide (CTAB) and phenol-chloroform mixture (phenol-chloroform-isoamyl alcohol, 25:24:1) and via the spin column (from the SanPrep column DNA gel extraction kit; Sangon Biotech, China) with the same method outlined by Zhang and colleagues (20). A NanoDrop ND-2000 UV spectrophotometer (Thermo Fisher Scientific, Carlsbad, CA) was used to check DNA concentration and DNA quality (*A*_260_/*A*_280_). DNAs with an *A*_260_/*A*_280_ ratio of 1.8 to 2.0 were used for PCR amplification. For sequencing DNA from fecal samples, the 2-step PCR was run with two proposed universal primers of V3-V4 region of 16S rRNA gene, forward primer-341F (TTCCCTACACGACGCTCTTCCGATCT XXXXXX CCTACGGGNGGCWGCAG) and reverse primer-805R (TTCCCTACACGACGCTCTTCCGATCT XXXXXX GACTACHVGGGTATCTAATCC) (the first segment refers to Illumina core sequence, XXXXXX refers to barcode, and a single underline refers to V3-V4 universal primer sequences). A 20-μl PCR mixture was as follows: 2 μl template DNA, 1 μl amplicon PCR forward primer (10 μM), 1 μl amplicon PCR reverse primer (10 μM), and 16 μl 2× *Taq* PCR mastermix. PCR was run with the program following: 1 cycle of denaturing at 94°C for 3 min, 6 cycles of denaturing at 94°C for 20 s, annealing at 55°C for 30 s, and elongation at 72°C for 30 s, then followed by 30 cycles of denaturing at 94°C for 15 s, annealing at 68°C for 15 s, elongation at 72°C for 20 s, and a final extension at 72°C for 5 min. The PCR products were purified by kit (GE0101-200; TsingKe Biological Technology, China), and then sequenced on an Illumina HiSeq 2500 platform (46).

The paired-end sequence data were joined and the quality was filtered using the FLASH method (47). All sequence analysis was done using QIIME (v1.9.1) according to the tutorial (http://qiime.org/) with some modifications (48). usearch61 with *de novo* models was used to remove chimeric sequences (49). Sequences were clustered against the 2013 Greengenes (13_8 release) ribosomal database’s 97% reference data set. The sequences that did not match any entries in this reference database were clustered with UCLUST into *de novo* operation taxonomic units (OTUs) at 97% similarity. Taxonomy was assigned to all OTUs using the RDP classifier within QIIME and the Greengenes reference data set (50). Rarefaction and rank abundance curves were calculated from OTU tables using α-diversity and rank abundance scripts within the QIIME pipeline. The hierarchical clustering based on population profiles of the most common and abundant taxa was performed using unweighted pair group method with arithmetic mean (UPGMA) clustering (also known as average linkage) on the distance matrix of OTU abundance. This resulted in a Newick-formatted tree, which was obtained utilizing the QIIME package.

### Statistical analysis.

During the course of intermittent acclimation to 5°C, 37°C, and 23°C conditions, data for body mass and *T_b_* were analyzed by repeated-measures analysis of variance (ANOVA), and food intake was analyzed by repeated-measures analysis of covariance (ANCOVA) with body mass as a covariate. Data for RMR and propionic acid on any time points of intermittent acclimation were examined by one-way ANCOVA or ANOVA, followed by least-significant difference (LSD) *post hoc* tests when the main effects were significant. In experiments 2, DEI, digestibility, serum T3 and T4, leptin, and ghrelin were analyzed using one-way ANOVA, followed by LSD *post hoc* tests. For statistical analysis, we used SPSS Statistics 17.0 for windows (Chicago, IL). Data are presented as mean ± standard error of the mean (SEM), and *P* < 0.05 was considered statistical difference.

For appraisal of richness and diversity of bacteria, α-diversity was calculated by Chao1, observed OTUs, Shannon index, and PD whole-tree analysis (20, 51). Significant group differences in bacterial relative abundances were examined by one-way ANOVA. Principal coordinate analysis (PCoA; β-diversity) based on Bray-Curtis distance between the samples was made for the 3 groups during the 3 acclimation periods (weeks 4, 8, and 12) (46, 52, 53), and the significance for PCoA was tested with multivariate permutation tests using the nonparametric method ANOSIM (52). The linear discriminant analysis (LDA) effect size (LEfSe) method was performed by the computational tool, using the Kruskal-Wallis (KW) rank sum test on classes, the pairwise Wilcoxon test between subclasses of different classes, and the LDA on the relevant features, to identify the differential biomarkers (54). Bootstrapping (“-permutations 1000”) and Pearson correlation were used to calculate the correlation between OTUs and physiological parameters. The level of statistical significance was set at a *P* value of <0.05 (false-discovery rate-corrected *P* value). The graphics and statistics were developed in Excel and STAMP v2.1.3. (http://kiwi.cs.dal.ca/Software/STAMP).

### Data availability.

Raw sequence data have been deposited in the NCBI Sequence Read Archive under the accession number PRJNA662422.
