# Supplementary material for: Gut Microbiota and Host Thermoregulation in Response to Ambient Temperature Fluctuations
Source: mSystems. 2020 Oct 20;5(5):e00514-20. doi: 10.1128/mSystems.00514-20 (PMC7577294; doi:10.1128/mSystems.00514-20)
Supplement: TABLE S1 [file mSystems.00514-20-st001.docx]

| Time (weeks) | 2 | 4 | 6 | 8 | 10 | 12 |
| --- | --- | --- | --- | --- | --- | --- |
| Acetic acid |  |  |  |  |  |  |
| C | 1254±125.8 | 1351±74.2 | 1302±80.9 | 1394±83.9 | 1511±108.5 | 1214±82. 7 |
| HC | 1101±80.3 | 1230±132.6 | 1289±88.8 | 1105±74.4 | 1202±120.9 | 1091±100.3 |
| LC | 1639±232.0 | 1460±129.8 | 1243±79.7 | 1339±122.0 | 1449±153.2 | 1533±226.0 |
| *F* | 3.408 | 0.967 | 0.151 | 1.644 | 1.274 | 1.854 |
| *P* | 0.062 | 0.396 | 0.86 | 0.22 | 0.301 | 0.183 |
| Propionic acid |  |  |  |  |  |  |
| C | 205.60±58.56 | 181±22.1^a^ | 1842±26.1 | 177±17.0^a^ | 184±22.4 | 159±19.6^ab^ |
| HC | 157.72±13.60 | 116±20.0^a^ | 137±13.9 | 95±4.5^b^ | 198±25.0 | 128±36.2^a^ |
| LC | 270.67±42.15 | 268±34.2^b^ | 201±23.2 | 234±23.0^c^ | 217±19.4 | 251±30.3^b^ |
| *F* | 3.635 | 7.622 | 2.232 | 10.859 | 0.64 | 5.02 |
| *P* | 0.054 | 0.003 | 0.133 | 0.001 | 0.538 | 0.017 |
| Isobutyric acid |  |  |  |  |  |  |
| C | 180±38.7 | 193±25.8 | 151±20.5 | 207±23.6 | 202±37.9 | 172±21.4 |
| HC | 214±21.6 | 176±18.9 | 233±50.7 | 183±24.2 | 155±14.8 | 147±21.8 |
| LC | 211±48.8 | 159±25.3 | 180±20.3 | 192±17.6 | 229±32.6 | 163±16.8 |
| *F* | 0.176 | 0.528 | 1.562 | 0.28 | 1.226 | 0.366 |
| *P* | 0.841 | 0.598 | 0.234 | 0.759 | 0.315 | 0.698 |
| Butyric acid |  |  |  |  |  |  |
| C | 115±13.1^ab^ | 113±22.8 | 104±27.2 | 100±8.8 | 198±49.1 | 108±21.3 |
| HC | 90±10.7^a^ | 109±31.7 | 79±14.4 | 48±7.0 | 141±41.7 | 53±15.7 |
| LC | 259±76.9^b^ | 202±40.2 | 121±14.2 | 132±26.0 | 132±25.4 | 149±50.1 |
| *F* | 3.909 | 2.616 | 1.287 | 4.006 | 0.876 | 1.536 |
| *P* | 0.045 | 0.097 | 0.298 | 0.035 | 0.432 | 0.24 |
| Isovaleric acid |  |  |  |  |  |  |
| C | 4.31±0.85 | 3.30±0.42 | 3.57±0.57 | 4.98±1.06 | 5.44±1.23 | 4.50±0.76 |
| HC | 3.56±0.53 | 3.57±0.96 | 2.77±0.49 | 5.68±0.89 | 4.35±0.42 | 4.04±0.79 |
| LC | 6.46±1.39 | 4.23±0.67 | 3.37±0.63 | 4.14±0.49 | 6.57±1.39 | 4.47±1.31 |
| *F* | 2.613 | 0.506 | 0.47 | 0.796 | 0.783 | 0.051 |
| *P* | 0.109 | 0.61 | 0.632 | 0.466 | 0.471 | 0.951 |
| Valeric acid |  |  |  |  |  |  |
| C | 27.62±3.07^ab^ | 22.51±2.37 | 19.42±2.17 | 23.39±3.63 | 32.86±6.72 | 29.06±5.50 |
| HC | 17.37±1.01^a^ | 20.80±3.16 | 18.33±2.22 | 21.61±3.17 | 21.87±3.30 | 18.04±3.55 |
| LC | 44.99±8.67^b^ | 28.38±5.24 | 22.07±2.76 | 23.95±3.23 | 30.79±4.50 | 20.55±4.08 |
| *F* | 7.74 | 1.013 | 0.632 | 0.101 | 1.05 | 1.51 |
| *P* | 0.005 | 0.38 | 0.542 | 0.904 | 0.368 | 0.245 |
