# Supplementary figures and images for: Gut Microbiota and Host Thermoregulation in Response to Ambient Temperature Fluctuations
Source: mSystems. 2020 Oct 20;5(5):e00514-20. doi: 10.1128/mSystems.00514-20 (PMC7577294; doi:10.1128/mSystems.00514-20)

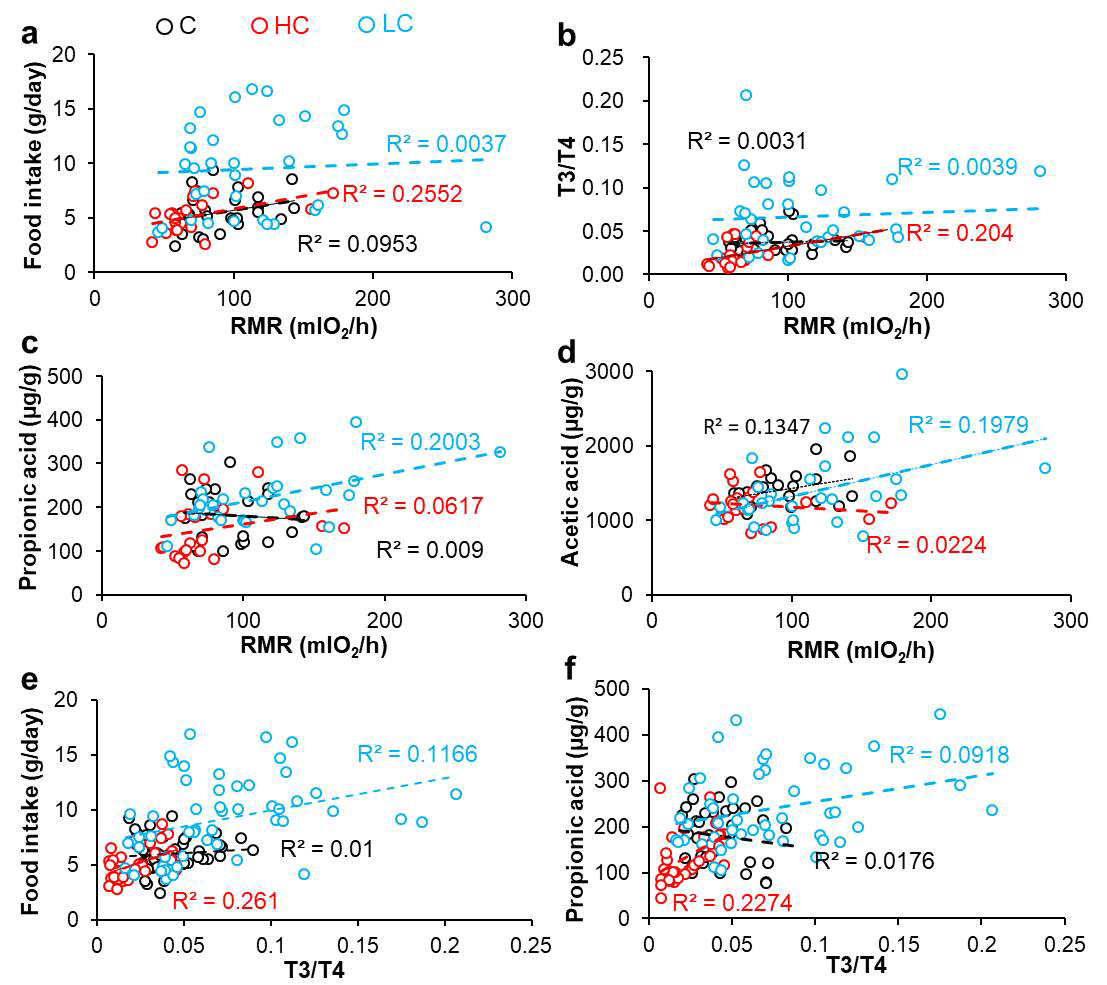

Supplement: FIG S1 [file mSystems.00514-20-sf001.tif]

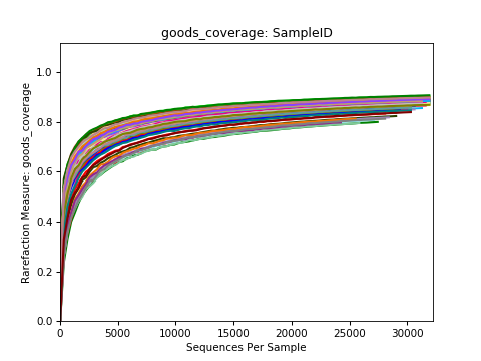

Supplement: FIG S2 [file mSystems.00514-20-sf002.tif]

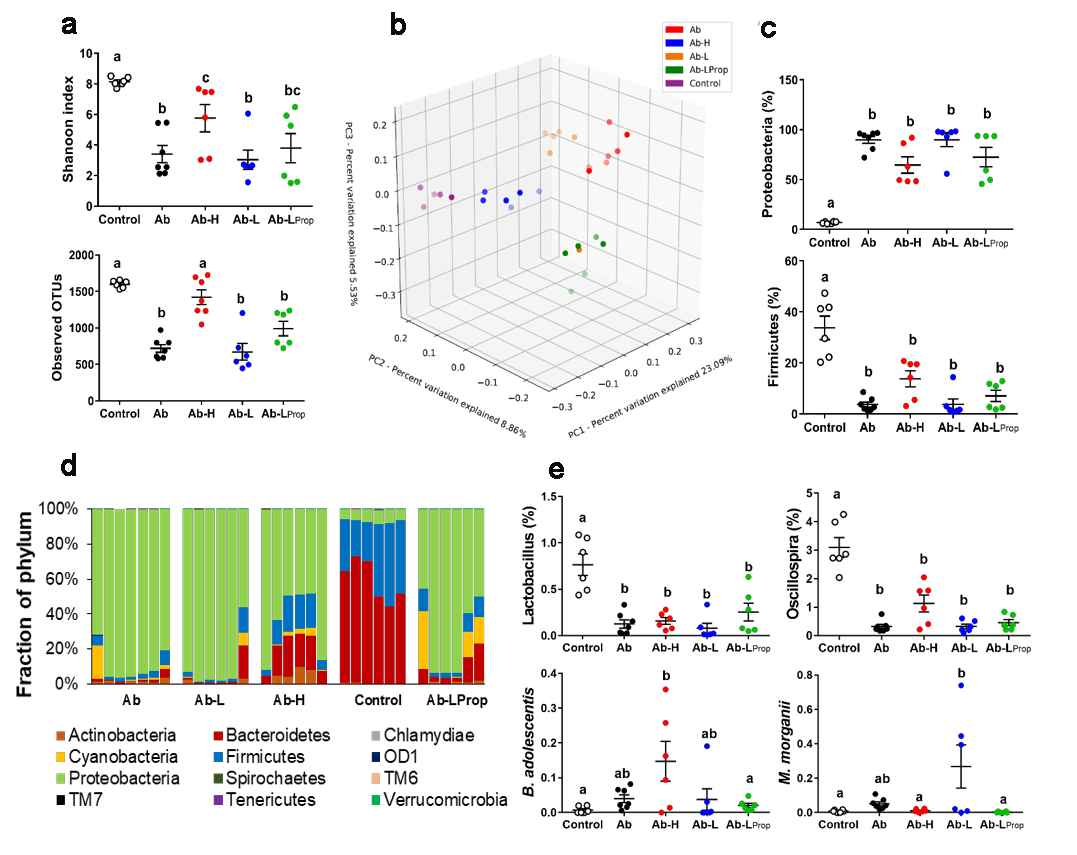

Supplement: FIG S3 [file mSystems.00514-20-sf003.tif]
