# Supplementary material for: Gut Microbiota and Host Thermoregulation in Response to Ambient Temperature Fluctuations
Source: mSystems. 2020 Oct 20;5(5):e00514-20. doi: 10.1128/mSystems.00514-20 (PMC7577294; doi:10.1128/mSystems.00514-20)
Supplement: TABLE S2 [file mSystems.00514-20-st002.docx]

|  | Body mass | Food intake | T3/T4 | RMR | Propionic acid | Acetic acid | Isobutyric acid | Butyric acid | Isovaleric acid | Valeric acid |
| --- | --- | --- | --- | --- | --- | --- | --- | --- | --- | --- |
| Body mass | 1 | 0.192 | -0.042 | .511** | 0.129 | 0.082 | -0.131 | 0.045 | -0.243 | -0.154 |
| Food intake | 0.192 | 1 | 0.205 | 0.309 | -0.031 | 0.252 | -0.031 | -0.06 | -0.073 | 0.023 |
| T3/T4 | -0.042 | 0.205 | 1 | 0.056 | -0.133 | -0.166 | -0.035 | -0.071 | -0.08 | -0.023 |
| RMR | .511** | 0.309 | 0.056 | 1 | -0.095 | 0.367 | 0.146 | -0.042 | -0.047 | 0.167 |
| Propionic acid | 0.129 | -0.031 | -0.133 | -0.095 | 1 | .533** | 0.215 | .519** | 0.188 | 0.261 |
| Acetic acid | 0.082 | 0.252 | -0.166 | 0.367 | .533** | 1 | .386* | .554** | .395* | .539** |
| Isobutyric acid | -0.131 | -0.031 | -0.035 | 0.146 | 0.215 | .386* | 1 | .325* | .529** | .494** |
| Butyric acid | 0.045 | -0.06 | -0.071 | -0.042 | .519** | .554** | .325* | 1 | .458** | .650** |
| Isovaleric acid | -0.243 | -0.073 | -0.08 | -0.047 | 0.188 | .395* | .529** | .458** | 1 | .785** |
| Valeric acid | -0.154 | 0.023 | -0.023 | 0.167 | 0.261 | .539** | .494** | .650** | .785** | 1 |
