# Supplementary material for: Gut Microbiota and Host Thermoregulation in Response to Ambient Temperature Fluctuations
Source: mSystems. 2020 Oct 20;5(5):e00514-20. doi: 10.1128/mSystems.00514-20 (PMC7577294; doi:10.1128/mSystems.00514-20)
Supplement: TABLE S3 [file mSystems.00514-20-st003.docx]

|  | Body mass | Food intake | T3/T4 | RMR | Propionic acid | Acetic acid | Isobutyric acid | Butyric acid | Isovaleric acid | Valeric acid |
| --- | --- | --- | --- | --- | --- | --- | --- | --- | --- | --- |
| Body mass (g) | 1 | 0.006 | 0.034 | 0.378 | 0.236 | -0.046 | 0.02 | 0.005 | -0.053 | 0.08 |
| Food intake | 0.006 | 1 | 0.511** | 0.505* | 0.485** | -0.002 | 0.096 | 0.019 | -0.103 | -0.041 |
| T3/T4 | 0.034 | 0.511** | 1 | 0.452 | 0.477** | 0.2 | 0.292 | 0.403* | -0.231 | 0.101 |
| RMR | 0.378 | 0.505** | 0.452** | 1 | 0.248 | -0.15 | -0.06 | 0.013 | -0.334 | -0.016 |
| Propionic acid | 0.236 | 0.485** | 0.477** | 0.248 | 1 | 0.383* | 0.156 | 0.509** | 0.164 | 0.463** |
| Acetic acid | -0.046 | -0.002 | 0.2 | -0.15 | 0.383* | 1 | 0.257 | 0.492** | 0.046 | 0.422** |
| Isobutyric acid | 0.02 | 0.096 | 0.292 | -0.06 | 0.156 | 0.257 | 1 | -0.21 | 0.001 | 0.159 |
| Butyric acid | 0.005 | 0.019 | 0.403* | 0.013 | .509** | 0.492** | -0.21 | 1 | 0.138 | 0.641** |
| Isovaleric acid | -0.053 | -0.103 | -0.231 | -0.334 | 0.164 | 0.046 | 0.001 | 0.138 | 1 | 0.594** |
| Valeric acid | 0.08 | -0.041 | 0.101 | -0.016 | 0.463** | 0.422** | 0.159 | 0.641** | 0.594** | 1 |
