# Supplementary material for: Gut Microbiota and Host Thermoregulation in Response to Ambient Temperature Fluctuations
Source: mSystems. 2020 Oct 20;5(5):e00514-20. doi: 10.1128/mSystems.00514-20 (PMC7577294; doi:10.1128/mSystems.00514-20)
Supplement: TABLE S4 [file mSystems.00514-20-st004.docx]

|  | Body mass | Food intake | T3/T4 | RMR | Propionic acid | Acetic acid | Isobutyric acid | Butyric acid | Isovaleric acid | Valeric acid |
| --- | --- | --- | --- | --- | --- | --- | --- | --- | --- | --- |
| Body mass | 1 | 0.253 | -0.038 | 0.391* | 0.293* | 0.072 | -0.202 | 0.066 | 0.112 | 0.165 |
| Food intake | 0.253 | 1 | 0.344* | 0.061 | 0.194 | 0.156 | -0.177 | -0.009 | -0.056 | -0.161 |
| T3/T4 | -0.038 | 0.344* | 1 | 0.062 | 0.303* | 0.007 | -0.177 | 0.155 | -0.234 | -0.051 |
| RMR | 0.391* | 0.061 | 0.062 | 1 | 0.448* | 0.445* | -0.283 | 0.546** | 0.134 | 0.413* |
| Propionic acid | 0.293* | 0.194 | 0.303* | 0.448* | 1 | 0.604** | -0.045 | 0.774** | 0.151 | 0.538** |
| Acetic acid | 0.072 | 0.156 | 0.007 | 0.445* | 0.604** | 1 | 0.098 | 0.655** | 0.333* | 0.479** |
| Isobutyric acid | -0.202 | -0.177 | -0.177 | -0.283 | -0.045 | 0.098 | 1 | -0.022 | 0.327* | 0.347* |
| Butyric acid | 0.066 | -0.009 | 0.155 | 0.546** | 0.774** | 0.655** | -0.022 | 1 | 0.253 | 0.680** |
| Isovaleric acid | 0.112 | -0.056 | -0.234 | 0.134 | 0.151 | 0.333* | 0.327* | 0.253 | 1 | 0.681** |
| Valeric acid | 0.165 | -0.161 | -0.051 | 0.413* | 0.538** | 0.479** | 0.347* | 0.680** | 0.681** | 1 |
