# Supplementary material for: Gut Microbiota and Host Thermoregulation in Response to Ambient Temperature Fluctuations
Source: mSystems. 2020 Oct 20;5(5):e00514-20. doi: 10.1128/mSystems.00514-20 (PMC7577294; doi:10.1128/mSystems.00514-20)
Supplement: TABLE S5 [file mSystems.00514-20-st005.docx]

| Time (weeks) | 2 | 4 | 6 | 8 | 10 | 12 |
| --- | --- | --- | --- | --- | --- | --- |
| Chao1 |  |  |  |  |  |  |
| C | 10143±327.7 | 10076±143.4 | 10042±362.5 | 11409±649.8 | 10219±483 | 10201±289.2^ab^ |
| HC | 9885±600.0 | 9674±277.1 | 9475±157.5 | 10430±380.7 | 10291±575 | 9478±303.7^a^ |
| LC | 10154±333.7 | 10062±162.5 | 9642±194.3 | 9682±469.2 | 10198±356 | 10963±300.3^b^ |
| *F* | 0.123 | 1.290 | 1.249 | 2.910 | 0.01 | 5.132 |
| *P* | 0.885 | 0.293 | 0.304 | 0.076 | 0.990 | 0.017 |
| Observed OTUs |  |  |  |  |  |  |
| C | 3676±90.8 | 3647±42.4 | 3599±104.1 | 4059±193.8 | 3612±119.9 | 3676±99.4 |
| HC | 3611±199.4 | 3509±106.0 | 3444±37.9 | 3757±121.5 | 3595±189.3 | 3438±94.1 |
| LC | 3689±88.0 | 3617±49.2 | 3489±49.9 | 3466±159.8 | 3619±120.4 | 3787±114.4 |
| *F* | 0.095 | 1.060 | 1.231 | 3.358 | 0.007 | 2.425 |
| *P* | 0.909 | 0.361 | 0.309 | 0.053 | 0.993 | 0.113 |
| Shannon Index |  |  |  |  |  |  |
| C | 9.40±0.13 | 9.37±0.04 | 9.45±0.12 | 9.70±0.19 | 9.50±0.16 | 9.45±0.09 |
| HC | 9.42±0.25 | 9.38±0.12 | 9.18±0.07 | 9.55±0.12 | 9.32±0.27 | 9.25±0.10 |
| LC | 9.52±0.09 | 9.35±0.06 | 9.11±0.10 | 9.24±0.18 | 9.41±0.12 | 9.50±0.15 |
| *F* | 0.178 | 0.031 | 2.946 | 1.729 | 0.212 | 0.941 |
| *P* | 0.838 | 0.969 | 0.07 | 0.203 | 0.811 | 0.406 |
| PD whole tree |  |  |  |  |  |  |
| C | 157±3.8 | 157±1.3 | 156±1.9 | 172±6.9 | 159.03±4.3 | 164±3.5^ab^ |
| HC | 157±7.1 | 156±3.5 | 150±2.3 | 167±5.2 | 163.08±7.8 | 157±2.9^a^ |
| LC | 157±3.8 | 157±1.7 | 153±2.2 | 155±5.4 | 161.26±4.3 | 171±5.5^b^ |
| *F* | 0.002 | 0.116 | 1.457 | 2.315 | 0.133 | 2.427 |
| *P* | 0.998 | 0.891 | 0.253 | 0.123 | 0.876 | 0.114 |
