# Supplementary material for: Gut Microbiota and Host Thermoregulation in Response to Ambient Temperature Fluctuations
Source: mSystems. 2020 Oct 20;5(5):e00514-20. doi: 10.1128/mSystems.00514-20 (PMC7577294; doi:10.1128/mSystems.00514-20)
Supplement: TABLE S6 [file mSystems.00514-20-st006.docx]

|  | DEI (kJ/day) | Digestibility (%) | T3 (ng/mL) | T4 (ng/mL) | Leptin (mg/mL) | Ghrelin (pg/mL) |
| --- | --- | --- | --- | --- | --- | --- |
| Control | 111.76±7.13^ab^ | 82.81±0.61^a^ | 0.67±0.05^bc^ | 32.41±3.02^a^ | 1.61±0.27^a^ | 230.04±14.76a |
| Ab | 87.06±7.90^b^ | 75.59±0.97^b^ | 0.46±0.05^ab^ | 16.55±1.57^bc^ | 1.24±0.49^ab^ | 103.55±27.67^b^ |
| Ab-H | 26.11±3.02^c^ | 67.57±3.92^b^ | 0.20±0.05^d^ | 8.45±1.00^d^ | 0.28±0.04^c^ | 41.34±5.92^c^ |
| Ab-L | 149.97±17.85^c^ | 72.90±2.76^b^ | 0.92±0.16^c^ | 18.63±1.58^b^ | 0.41±0.10^bc^ | 113.89±25.48^b^ |
| Ab-L_Prop_ | 118.99±5.98^bc^ | 72.06±1.55^b^ | 0.67±0.13^bc^ | 19.58±2.47^b^ | 0.94±0.36^abc^ | 68.17±17.98^bc^ |
| *F* | 25.312 | 5.641 | 10.706 | 20.896 | 4.477 | 14.780 |
| *P* | <0.001 | 0.002 | <0.001 | <0.001 | 0.008 | <0.001 |
