# Supplementary material for: Gut Microbiota and Host Thermoregulation in Response to Ambient Temperature Fluctuations
Source: mSystems. 2020 Oct 20;5(5):e00514-20. doi: 10.1128/mSystems.00514-20 (PMC7577294; doi:10.1128/mSystems.00514-20)
Supplement: TABLE S7 [file mSystems.00514-20-st007.docx]

|  | Chao1 | Observed OTUs | Shannon Index | PD whole tree |
| --- | --- | --- | --- | --- |
| Control | 2031±14.2^a^ | 1600±20.8^a^ | 8.14±0.12^a^ | 87.8±1.14^a^ |
| Ab | 1255±51.0^bc^ | 719±54.5^b^ | 3.40±0.56^b^ | 52.2±3.15^b^ |
| Ab-H | 2177±87.9^a^ | 1450±113.2^a^ | 5.76±0.90^c^ | 85.8±5.03^a^ |
| Ab-L | 1184±126.8^c^ | 672±114.0^b^ | 3.04±0.63^b^ | 50.7±5.80^b^ |
| Ab-L_Prop_ | 1570±104.0^b^ | 996±100.7^b^ | 3.79±0.95^bc^ | 67.7±5.02^b^ |
| *F* | 27.96 | 23.50 | 9.40 | 17.09 |
| *P* | <0.001 | <0.001 | <0.001 | <0.001 |
